# O.5.1-6 Bucking the mid-life inactivity trend: a study of 40–64-year-olds' participation in Zwift, a real-time online exercise community, through a social identity lens

**DOI:** 10.1093/eurpub/ckad133.236

**Published:** 2023-09-11

**Authors:** Toby Richards, Sean Figgins, Melissa Day, Matthew Slater, Matthew Easterbrook

**Affiliations:** University of Sussex, UK; University of Sussex, UK; University of Chichester, United Kingdon; Staffordshire University; tr345@sussex.ac.uk; University of Sussex, UK

## Abstract

**Purpose:**

Despite evidence suggesting a decline in the physical activity of middle-aged adults, 40–64-year-olds participation in group exercise is actually increasing. One potential reason for this increase in activity is the increase in a range of different online group exercise formats and contexts. Recent research suggests that people join, participate, and stay involved in, exercise groups that reflect who they are - a process of social identification. The purpose of this research is to qualitatively explore participants’ experiences and facilitators of social identification related to the online group exercise platform known as Zwift.

**Methods:**

Zwift participants (n = 17) aged between 40-64 (midlife) (13 Men, 4 Women) were recruited into the study for three stages of data collection: (stage 1) an initial semi-structured interview exploring participant exercise history and use of Zwift, (stage 2) completing a two-week post exercise diary to capture their experiences of social identification after participation, (stage 3) a follow up final interview to explore topics raised over the first two data collection steps. This generated 23 hours of interviews, that were then transcribed and combined with the participant diaries resulting in 468 pages of data.

**Results:**

Reflexive thematic analysis was used to identify themes from the data. The results from this analysis indicate the following themes from the data: (1) Online exercise as an enabling facilitator of exercise identity; (2). Online exercise provides exercise identity continuity or exercise identity creation depending on the exercisers, exercise history; (3) Online exercise identification via communities goes through phases of identification; (4) Two-way communication contributes to perceptions of identification phases of identification; and (5) Social identity leadership is demonstrated by online exercisers norms, behaviours and beliefs

**Conclusions:**

Overall, this research highlights three key suggestions for mid-life physical activity promotion: (1) That facilitators and processes of social identification may be context specific; (2) That social identification processes should be considered in the design of online exercise; and (3) That online exercise that enables social identification should be considered as a vehicle of maintaining/ increasing exercise participation

**Funding:**

The study was funded by the researcher.

